# The role of librarians in teaching evidence-based medicine to pediatric residents: a survey of pediatric residency program directors

**DOI:** 10.5195/jmla.2017.178

**Published:** 2017-10-01

**Authors:** Rachel Boykan, Robert M. Jacobson

## Abstract

**Objective:**

The research sought to identify the general use of medical librarians in pediatric residency training, to define the role of medical librarians in teaching evidence-based medicine (EBM) to pediatric residents, and to describe strategies and curricula for teaching EBM used in pediatric residency training programs.

**Methods:**

We sent a 13-question web-based survey through the Association of Pediatric Program Directors to 200 pediatric residency program directors between August and December 2015.

**Results:**

A total of 91 (46%) pediatric residency program directors responded. Most (76%) programs had formal EBM curricula, and more than 75% of curricula addressed question formation, searching, assessment of validity, generalizability, quantitative importance, statistical significance, and applicability. The venues for teaching EBM that program directors perceived to be most effective included journal clubs (84%), conferences (44%), and morning report (36%). While 80% of programs utilized medical librarians, most of these librarians assisted with scholarly or research projects (74%), addressed clinical questions (62%), and taught on any topic not necessarily EBM (58%). Only 17% of program directors stated that librarians were involved in teaching EBM on a regular basis. The use of a librarian was not associated with having an EBM curriculum but was significantly associated with the size of the program. Smaller programs were more likely to utilize librarians (100%) than were medium (71%) or large programs (75%).

**Conclusions:**

While most pediatric residency programs have an EBM curriculum and engage medical librarians in various ways, librarians’ expertise in teaching EBM is underutilized. Programs should work to better integrate librarians’ expertise, both in the didactic and clinical teaching of EBM.

## INTRODUCTION

The practice of medicine requires that physicians remain current in their knowledge of the literature. Twenty years ago, Sackett et al. defined evidence-based medicine (EBM) as “the conscientious, explicit and judicious use of current best evidence in making decisions about the care of the individual patient” [1]. Today, the Accreditation Council for Graduate Medical Education requires that pediatric residents have training in EBM. Pediatric residents “must demonstrate the ability to investigate and evaluate their care of patients, to appraise and assimilate scientific evidence” [2]. However, how such training is defined and the venue in which it is taught varies widely and may include journal clubs, workshops, conferences, lectures, morning reports, and integration into clinical rounds. Medical librarians have long been recognized as a resource for practicing physicians, but only more recently has their expertise been used in training physicians in search strategies and evaluation of the literature [3–8].

Librarians have provided valuable input in collaborations with physicians in the design and implementation of medical school and residency curricula [9, 10]. At the University of Michigan, librarians partnered with deans of graduate medical education to create a curriculum to teach information-gathering skills to address the “Problem Based Learning and Improvement” competency [11]. At Stony Brook University, pediatric faculty collaborated with a medical librarian in the creation of a 3-year longitudinal curriculum for pediatric residents [7, 12, 13].

Such collaboration between librarians and physicians in teaching and practicing EBM has been shown to impact patient care. In one recent study by Aitken et al., librarians’ participation in a patient care team had a positive effect on medical residents’ and students’ self-reported ability to locate and evaluate evidence related to patient care compared with a control team with no librarian presence. In that study, 88% (30/34) of respondents reported changing a treatment plan based on skills learned from librarians, and 79% (27/34) reported changing a treatment plan based on direct searching support by the librarian [5]. Another case control study compared morning report presentations with a librarian present to those with no librarian present. Librarians provided input and literature reviews relevant to the clinical case, resulting in shortened patient length of stay and lower hospital charges compared with controls [14]. In another example, librarians functioned as consultants, providing “just in time” literature searches for clinical questions in the primary care setting [15].

Using a web-based survey sent through the Association of Pediatric Program Directors (APPD) to all member pediatric program directors, the authors sought to (1) identify the general use of medical librarians in pediatric residency training, (2) define the role of medical librarians in teaching EBM to pediatric residents, and (3) describe strategies and curricula for teaching EBM in pediatric residency training programs.

## METHODS

We developed a 13-question electronic survey utilizing multiple-choice and short-answer question formats regarding the use of medical librarians in pediatric residency programs. A medical librarian provided input in the initial development and refinement of the survey. Volunteer members of the Academic Pediatric Association Evidence-Based Pediatrics Special Interest Group piloted drafts of the survey. The APPD Research and Scholarship Task Force reviewed and approved the final version of the survey for distribution as an officially approved APPD survey (supplemental appendix).

The APPD reports that, at the time of this survey, there were 220 pediatric residency programs in the United States, of which 200 were APPD members. Between August and December 2015, we sent all 200 APPD-member pediatric residency program directors a link to our web-based survey at SurveyMonkey [16]. Consent was implied by completion of the survey. Up to 6 reminders were sent to nonrespondents.

We collected demographic information including program name, geographic region, and program size. We then asked respondents if their residency programs utilized medical librarians. For respondents who indicated that their programs utilized librarians, we inquired about the specific role of librarians, including their integration into EBM curricula. For respondents who indicated that their programs did not utilize librarians, we inquired about reasons for not utilizing librarian services and asked program directors to describe their EBM curricula. For some analyses, the 8 APPD regions were combined to form 4 regions: Mid-American and Midwest; Southeast; West and Southwest; and Mid-Atlantic, Northeast, and New York. Both Stony Brook University Medical Center’s and Mayo Clinic’s institutional review boards approved the study. We used Statistical Package for the Social Sciences (SPSS), version 22, software for descriptive, cross-tabular, and logistic regression analysis, defining statistical significance as a 2-tailed *p*<0.05.

## RESULTS

### Demographics

A total of 91 (46%) APPD-member program directors responded. As shown in Table 1, for each of the 8 regions, response rates ranged from 22%–73%, with the median being 37.5%. The mean program size was 47 residents, with a range of 10–160 residents. Twenty-eight percent of respondents were from small programs (<30 residents total), 45% from medium-sized programs (30–59 residents), and 27% from large programs (≥60).

**Table 1:** Survey participation by region

**Region**	**Pediatric residency programs participating in survey**	**Total number of pediatric residency programs**	**Percent of pediatric residency programs participating in survey**
Mid America (western PA, OH, WV, KY, IN, MI)	6	27	22%
Mid Atlantic (southern NJ, eastern PA, DE, MD, DC)	9	20	45%
Midwest (IL, WI, MN, IA, MO, KS, NE, OK, SD)	22	30	73%
New England (ME, NH, MA, CT, VT, RI)	8	13	62%
New York (NY, northern NJ)	12	36	33%
Southeast (VA, NC, SC, GA, FL, AL, MS, LA, AR, TN)	19	51	37%
Southwest (TX)	5	13	38%
Western (CA, NV, OR, WA, AK, CO, NM, UT, AZ, HI)	10	30	33%
Total	91	220	41%

### Evidence-base medicine (EBM) curriculum

Seventy-six percent of program directors had a formal EBM curriculum in their residency programs. Because much of resident learning occurs in the context of clinical experience, we defined “formal” as any EBM learning experienced by design, rather than by ad-hoc or unplanned teaching.

As shown in Table 2, more than three-fourths of the programs taught using population, intervention, comparator, outcome (PICO) question formation; using search strategies; locating clinically relevant articles, assessing validity, assessing generalizability, evaluating quantitative importance, evaluating results for statistical significance, and appraising applicability. Forty-one percent of programs taught how to create a critically appraised topic (CAT), which is “a standardized summary of research evidence organized around a clinical question, aimed at providing both a critique of the research and a statement of the clinical relevance of results” [17].

**Table 2:** Evidence-based medicine (EBM) skills taught within residency program (n=91)

**Skill**	
Search literature	92%
Appraise study’s applicability to patient care	86%
Assessing study’s validity	81%
Evaluate quantitative importance (e.g., effect size, number needed to treat)	81%
Evaluate statistical significance	81%
Frame population, intervention, comparator, outcome (PICO) question	78%
Locate studies applicable to patient care	78%
Assess study’s generalizability	76%
Create a critically appraised topic (CAT)	41%
Other	4%

As indicated in Table 3, respondents identified that the 3 most effective venues through which EBM concepts were taught in their residency program were journal clubs (84%), regularly occurring EBM conferences or seminars (44%), and morning reports (36%). Other learning opportunities included lectures, occasional workshops, clinical rounds, attending rounds, clinical precepting, self-learning, case reports, and chief resident rounds. Table 4 lists the individuals in the programs who were responsible for teaching EBM. Other attending physicians (96%) and the program director (86%) were most frequently responsible, whereas medical librarians were responsible in 37% of the programs.

**Table 3:** Perception of most effective venues for teaching EBM concepts (n=91)

**Venue**	
Journal club	84%
Regular EBM conferences or seminars	44%
Morning report	36%
Lectures	31%
Occasional workshops	21%
Clinical rounds	15%
Attending rounds	12%
Clinical precepting	11%
Self-learning	11%
Other (e.g., online modules, team-based learning, individualized curriculum)	8%
Case reports	6%
Chief resident rounds	1%

**Table 4:** Individuals responsible for teaching EBM to residents (n=91)

**Role**	
Other attending physicians	96%
Program director	86%
Chief residents	37%
Medical librarians	37%
Residents	32%
Other (epidemiologist, biostatistician, associate program director)	9%
Medical students	1%

### Role of medical librarians in pediatric residency programs

While medical librarians were identified as responsible for teaching EBM in 37% of programs, 74 program directors (81%) stated that their programs utilized medical librarians in some way in training pediatric residents. Common librarian roles included assisting with scholarly or research projects (74%), addressing clinical questions as they occurred (62%), and teaching a seminar or a conference on any topic (not necessarily EBM) (58%). A minority of programs included librarian participation in an EBM journal club (22%) or on clinical rounds (17%). Seventeen percent of program directors stated that librarians were involved in teaching EBM on a regular basis. Librarians were involved with curriculum development in 8% of programs.

The mean number of years that programs had utilized librarians as recalled by the program director was 11, with a range of 1 to 35. Almost half of the programs had utilized librarians for 1 to 9 years (48.6%); 32% had utilized librarians for 10 to 19 years; and 19.4% had utilized librarians for 20 to 35 years. The majority of program directors reported that there was no monetary charge to the residency program for librarians’ services: 57.0% were paid through the hospital or health care system, 40.0% through the medical school, and 3.9% through a line-item budget charged to the residency program. Some (10.4%) respondents did not know how the librarian was paid.

Eighteen program directors (20%) stated their programs did not utilize librarians. Reasons given were lack of awareness of medical librarians’ availability (8/18; 44%), limited availability of librarians (9/18; 50%), lack of interest on the part of the program (9/18; 50%), lack of resources (4/18; 22%), lack of librarians’ knowledge of EBM processes (4/18; 22%), or previous experience with medical librarians did not warrant continued usage (1/18; 5%).

Cross-tabular analysis revealed that having a formal curriculum to teach EBM was not associated with utilizing a librarian. However, utilizing a medical librarian was significantly associated with the size of the program: smaller programs were more likely to utilize librarians (100%) than were medium (71%) or large programs (78%) (χ^2^=9.369; *df*=2; *p*=0.009; Table 5).

**Table 5:** Librarian usage by program size (n=91)

	**Respondents**	**Librarian usage**	
	
	**n**	**n**	**(%)**
Small programs (≤29 residents)	27	27	(100%)
Medium programs (30–59 residents)	41	29	(71%)
Large programs (>60 residents)	23	18	(78%)

## DISCUSSION

In our survey, 74% of pediatric residency programs had an EBM curriculum in place, whereas Kersten et al.’s survey of pediatric chief residents found that only 48% of programs had a formal EBM curriculum 12 years ago [18]. In both Kersten’s study and our study, programs utilized a number of venues for teaching EBM and covered a broad range of topics, including framing a PICO question, searching the literature, and critically appraising and applying the literature. Another similarity between Kersten’s study and our study was that both identified the same venues as the most effective for teaching EBM: journal clubs, workshops or conferences, and morning report [18]. However, the integration of EBM learning into a clinical context (clinical rounds, clinical precepting, attending rounds) was not highly rated by our respondents, which might reflect a narrow definition of EBM focusing on critical appraisal skills. However, the importance of learning EBM skills in a clinically relevant context has been highlighted by several studies [13, 19]. For example, at one institution, the use of tablets at the bedside improved EBM knowledge, skills, and behavior [19]. In a recent systematic review, common barriers to resident education in EBM were limited time, attitude, knowledge, and skills [20]. Integrating EBM education more broadly into a residency program would address these gaps.

A majority of pediatric residency programs utilized the expertise of medical librarians, most commonly for clinical or research help, but fewer than 50% of programs utilized librarians for EBM training. This might be explained, in part, by the predominance of journal clubs as the main forum for EBM teaching, in which the librarian might not be seen as having a significant role. However, we found it interesting that 91% of respondents stated that residents learned searching skills in their EBM curricula. Does this mean that faculty are teaching these skills? Are these skills being taught effectively? Several studies have demonstrated the importance of the librarians’ role in teaching EBM by providing direct instruction or a “train the trainer” approach [21, 22].

Smaller pediatric residency programs were significantly more likely to use librarians than medium or large programs. Although the reasons were not entirely clear, this finding perhaps reflected a relative lack of resources for small programs. Regardless, for all program sizes, the cost of utilizing librarians did not appear to be a barrier.

Some limitations of this study should be mentioned. With a 46% response rate, our data might not be fully representative of current EBM teaching practices, although we had a proportionate distribution of regions and program sizes. We assumed that program directors completed the survey themselves, as it was directly emailed to them; however, it was possible in some cases that they arranged for someone else to complete the surveys. The questionnaire was not a validated tool but was adapted from a previous study [18]. In its final version, the skip logic of question 8 was altered, and responses were recoded by hand by both authors. However, we do not feel this affected the validity of the answers. Selection bias might have been a factor in the program directors’ assessment of the most effective venues for teaching EBM if their programs were limited to only a few venues. Finally, the program directors’ evaluation of their own EBM programs’ effectiveness could be subject to reporter bias, especially, if the program directors were involved in the curriculum.

EBM is taught in a variety of venues in pediatric residency programs, but the integration of EBM education into the clinical arena is less well described. Medical librarians may be important in designing, implementing, and teaching EBM skills in pediatric residency programs but are likely underutilized. Program directors and faculty should work to better integrate librarians’ expertise, both in the didactic and clinical teaching of EBM.

## SUPPLEMENTAL FILE

Appendix Survey instrument
