# Supplementary material for: The role of librarians in teaching evidence-based medicine to pediatric residents: a survey of pediatric residency program directors
Source: J Med Libr Assoc. 2017 Oct 1;105(4):355–60. doi: 10.5195/jmla.2017.178 (PMC5624425; doi:10.5195/jmla.2017.178)
Supplement: Appendix [file jmla-105-355-s001.pdf]

JMLA Journal of the Medical Library Association

SC

Supplemental content to
J Med Libr Assoc. Oct;105(4): dx.doi.org/10.5195/jmla.2017.178
www.jmla.mlanet.org
© Boykan, Jacobson 2017

# The role of librarians in teaching evidence-based medicine to pediatric residents: a survey of pediatric residency program directors

Rachel Boykan, MD; Robert M. Jacobson, MD

## APPENDIX

### Survey instrument

Dear Subject,
You are being asked to volunteer in a research study, "Pediatric Resident Evidence-Based Medicine Education and the Librarian's Role (A Survey of US Pediatric Residency Program Directors)."

The purpose of the study is to identify if and how medical librarians are utilized in pediatric residency training and specifically to define the role of medical librarians in teaching evidence-based medicine (EBM) to pediatric residents.

If you agree to participate, your part will be to complete the online survey. It should take no more than 5–7 minutes of your time. There are no foreseeable risks or benefits to you for participating in this study. You will not be paid ($) for your participation.

The following procedures will be followed in an effort to keep your personal information confidential: data collected will remain confidential. To ensure that this research activity is being conducted properly, [RedactedRedacted] and/or applicable officials of [RedactedRedacted] have the right to review study records, but confidentiality will be maintained as allowed by law. There are no costs to you for participating in this study.

Your participation is completely voluntary. You do not have to participate if you do not want to. If you have any questions, concerns, or complaints about the study, you may contact me at [RedactedRedacted].

If you have any questions about your rights as a research subject, or if you would like to obtain information or offer input, you may contact [RedactedRedacted].

If you complete the attached survey, it means that you have read (or have had read to you) the information contained in this letter and would like to be a volunteer in this research study.

Thank you,

[RedactedRedacted]

**Your residency program description**

This survey should take you 5 to 7 minutes to complete, depending upon whether your program uses librarians. Thank you for your participation.

1. How many categorical pediatric residents are in your program (including all three years)?
Categorical pediatric residents ____________________

2. Please identify your residency program. (Note the program may be listed by the sponsoring organization and not the associated medical school.)

____________________

JMLA Journal of the Medical Library Association

SC
Supplemental content to
J Med Libr Assoc. Oct;105(4): dx.doi.org/10.5195/jmla.2017.178
www.jmla.mlanet.org
© Boykan, Jacobson 2017

3. Which of the following best describes your medical library? You may choose more than one answer.
- ☐ Associated with or part of a medical school
- ☐ Associated with or part of a university
- ☐ Part of a hospital or hospital system
- ☐ Other (please specify) ______________________

4. Does your residency program utilize medical librarians?
- ☐ Yes
- ☐ No

5. In which ways does your residency program routinely engage or staff medical librarians? (Check all that apply)
- ☐ Addressing clinical questions as they occur
- ☐ Teaching a seminar or conference
- ☐ Regularly participating in clinical rounds
- ☐ Regularly teaching evidence-based medicine (EBM)
- ☐ Regularly participating in an EBM journal club
- ☐ Curriculum development
- ☐ Assistance with scholarly/research projects
- ☐ Other (please specify) ______________________

6. How does your residency program pay for your use of the medical librarians?
- ☐ Line item budget charged to residency program
- ☐ Available through the medical school at no additional expense
- ☐ No charge (hospital or health care system is paying)
- ☐ Do not know
- ☐ Other (please specify) ______________________

7. For approximately how many years has your residency program been routinely engaging medical librarians?
Years: ______________________

**Resources**

8. If your program does not use medical librarians, which reasons limit your program's use of medical librarians?
- ☐ Lack of awareness of medical librarians' availability
- ☐ Lack of resources to pay for medical librarians' time
- ☐ Lack of medical librarians knowledgeable in EBM process
- ☐ Previous experience with medical librarians did not merit continued participation
- ☐ Limited availability of librarians
- ☐ Lack of interest on program's part
- ☐ Lack of interest on librarian's part
- ☐ Other (please specify) ______________________

9. Does your residency program have a formal curriculum to teach EBM?
- ☐ Yes
- ☐ No

JMLA Journal of the Medical Library Association

SC
Supplemental content to
J Med Libr Assoc. Oct;105(4): dx.doi.org/10.5195/jmla.2017.178
www.jmla.mlanet.org
© Boykan, Jacobson 2017

10. Which of the following EBM skills are taught in your residency program? (Check all that apply)
- ☐ How to frame a population, intervention, comparator, outcome (PICO) question from a clinical scenario
- ☐ How to search the literature using a clinical question
- ☐ How to assess a study's validity
- ☐ How to assess a study's generalizability
- ☐ How to evaluate a study's results for quantitative importance (i.e., effect size, number needed to treat [NNT], clinical importance)
- ☐ How to evaluate a study's results for statistical significance
- ☐ How to locate studies applicable to patient care
- ☐ How to appraise a study's applicability to patient care
- ☐ How to create a critically appraised topic (CAT)
- ☐ Other (please specify) ____________________

11. Identify up to the three most effective venues in which EBM concepts listed are taught in your residency program.
- ☐ Morning report
- ☐ Lectures
- ☐ Case reports
- ☐ Chief resident rounds
- ☐ Attending rounds
- ☐ Journal clubs
- ☐ Clinical rounds
- ☐ Clinical precepting
- ☐ Regularly occurring EBM conferences or seminars
- ☐ Occasional resident workshops on EBM
- ☐ Self-learning
- ☐ Other (please specify) ____________________

12. Which individuals are responsible for teaching EBM to the pediatric residents in your program? (check all that apply)
- ☐ Medical students
- ☐ Residents
- ☐ Chief resident(s)
- ☐ Program director
- ☐ Other attending(s)
- ☐ Medical librarians
- ☐ Other (please specify) ____________________

13. How satisfied are you with the quality of the EBM taught in your program?
- ☐ Very satisfied
- ☐ Somewhat satisfied
- ☐ Not satisfied

Thank you for completing our survey!
